# Synchronic historical patterns of species diversification in seasonal aplocheiloid killifishes of the semi-arid Brazilian Caatinga

**DOI:** 10.1371/journal.pone.0193021

**Published:** 2018-02-16

**Authors:** Wilson J. E. M. Costa, Pedro F. Amorim, José Leonardo O. Mattos

**Affiliations:** Laboratory of Systematics and Evolution of Teleost Fishes, Institute of Biology, Federal University of Rio de Janeiro, Rio de Janeiro, Rio de Janeiro, Brasil; Universita degli Studi di Roma La Sapienza, ITALY

## Abstract

The Caatinga is the largest nucleus of seasonally dry tropical forests in South America, but little is known about the evolutionary history and biogeography of endemic organisms. Evolutionary diversification and distribution of terrestrial vertebrates endemic to the Caatinga have been explained by palaeogeographical Neogene episodes, mostly related to changes in the course of the São Francisco River, the largest river in the region. Our objective is to estimate the timing of divergence of two endemic groups of short-lived seasonal killifishes inhabiting all ecoregions of the Caatinga, testing the occurrence of synchronic events of spatial diversification in light of available data on regional palaeogeography. We performed independent time-calibrated phylogenetic molecular analyses for two clades of sympatric and geographically widespread seasonal killifishes endemic to the Caatinga, the *Hypsolebias antenori* group and the *Cynolebias* alpha-clade. Our results consistently indicate that species diversification took place synchronically in both groups, as well as it is contemporary to diversification of other organisms adapted to life in the semi-arid Caatinga, including lizards and small mammals. Both groups originated during the Miocene, but species diversification started between the Late Miocene and Early Pliocene, when global cooling probably favoured the expansion of semi-arid areas. Synchronic diversification patterns found are chronologically related to Tertiary palaeogeographical reorganizations associated to continental drift and to Quaternary climatic changes, corroborating the recent proposal that South American biodiversity has been continuously shaped between the Late Paleogene and Pleistocene.

## Introduction

The Caatinga is a semi-arid phytogeographical province of north-eastern Brazil with an area of about 850,000 km^2^, representing the largest nucleus of seasonally dry tropical forests in South America [1]. In the past, it was considered as a low biodiversity region, but recent studies have consistently revealed a rich diversity of endemic plants and animals previously unknown for scientists [2, 3]. Continuous field studies in the last three decades directed to sample small temporary pools situated near rivers and streams of the Caatinga have demonstrated an unexpectedly high diversity of endemic seasonal killifishes [4, 5, 6]. Seasonal or annual killifishes [7, 8] are members of the cyprinodontiform suborder Aplocheiloidei occurring in tropical and subtropical areas of the Americas and Africa [9, 10, 11]. They have resistant eggs that undergo embryonic diapause during dry seasons, when pools disappear, and a new generation arises at every rainy season [12].

Evolutionary diversification of small terrestrial vertebrates endemic to the Caatinga has been explained by Neogene landscape changes, mostly related to gradual transformation of the course of the São Francisco River (SFR), the largest river in the region [3, 13]. These episodes include the uplift of large crystalline plateaus (*i*.*e*., Borborema-Araripe range) during the Miocene, isolating SFR from adjacent northeastern river basins (*i*.*e*., Piranhas and Jaguaribe); interruption of the lower SFR palaeocourse which was close or coincident with the present Parnaíba River course, separating the SFR and Parnaíba River basins at the Tertiary end; and, formation of a putative lower SFR palaeodrainage running to east and connected to river basins south of its present lower course (i.e., Vazabarris and Itapicuru river basins) during the Early Pleistocene, subsequently changing to the present course during the Middle Pleistocene [13, 14, 15, 16, 17].

Two not closely related killifish genera occur in the Caatinga, *Hypsolebias* Costa, 2006 and *Cynolebias* Steindachner, 1876 [18]. Among them, two species groups are remarkable by being geographically widespread along all the main biogeographical areas of the Caatinga, the *Hypsolebias antenori* group [5, 19, 20], hereafter HAG, and the *Cynolebias* alpha-clade [4], hereafter CAC. Species of both groups are found in sympatry, constituting the most common members of seasonal killifishes in temporary pools of the whole region. The 15 species of HAG considered valid are medium sized killifishes, reaching between about 45 and 75 mm of standard length (SL; measured between snout tip and caudal-fin base); they are highly sexually dimorphic, with males exhibiting striking colouration and filamentous dorsal and anal fins [5, 21]. The 16 valid species of CAC range from medium to large size, some reaching about 125 mm SL, standing among the largest aplocheiloid species [4]; they are the only aplocheiloids reported to produce sounds during courtship behaviour [22, 23] through a complex pharyngeal apparatus [24].

The great diversity of species, the broad area occupied by these groups and their specialized ecology make seasonal killifishes potential candidates to test evolutionary patterns of species diversification in the Caatinga. Costa [21, 25] noted some congruence in the distribution patterns of *Cynolebias* and *Hypsolebias*, concluding that the present distribution of both groups was shaped by the same vicariance events, but no timing estimate for their diversification was then available. We herein provide the first molecular phylogenetic analyses for representative samples of species of both groups. We use independent time-calibrated analyses to estimate the timing of divergence of lineages inhabiting different ecoregions of each group, testing the occurrence of synchronic events of spatial diversification in light of available data on regional palaeogeography.

## Materials and methods

### Ethics statement

This study was approved by the Ethics Committee for Animal Use of Federal University of Rio de Janeiro (CEUA-CCS-UFRJ, permit number: 01200.001568/2013-87) and was conducted according to national and international guidelines. Specimens were euthanized just after collection in a buffered solution of ethyl-3-amino-benzoat-methansulfonat (MS-222) at a concentration of 250 mg/l, for a period of 10 minutes or more, until completely ceasing opercular movements. Collections were made with permits provided by Instituto Chico Mendes de Conservação da Biodiversidade (ICMBio; permit numbers 34270–4, 20618–1, 57099–1).

### Taxon sampling

For the HAG analysis, we used 14 of the 15 species considered valid, and for the CAC analysis, we used 15 of the 18 species considered valid (species names, their respective collecting locality coordinates and GenBank accession numbers are listed in S1 Table); the only valid species not sampled were *Hypsolebias macaubensis* (Costa, 2006) and *Cynolebias microphthalmus* Costa, 1995, not found during recent field studies, in addition to two recently described but possibly extinct species, *C*. *elegans* Costa, 2017 and *C*. *gorutuba* Costa, 2017 [26]. In both analyses, out-group species were closely related members of the tribe Cynolebiini [18] (see S1 Table). All analyses were made through tissues deposited in the Institute of Biology, Federal University of Rio de Janeiro, Rio de Janeiro (UFRJ) of specimens previously collected in the field, between 2008 and 2010, or specimens collected later with small dip nets (40 X 30 cm), euthanized just after collection in a buffered solution of ethyl-3-amino-benzoat-methansulfonat (MS-222) at a concentration of 250 mg/l, for a period of 10 minutes or more, until completely ceasing opercular movements, fixed just after collection in absolute ethanol and later preserved in the same fixative; vouchers specimens are deposited in UFRJ. Collections were made with permits provided by ICMBio (Instituto Chico Mendes de Conservação da Biodiversidade).

### DNA extraction, amplification and sequencing

Total genomic DNA was extracted from muscle tissue of the right side of the caudal peduncle using the DNeasy Blood & Tissue Kit (Qiagen), according to the manufacturer instructions. For the HAG analysis, we used partial sequences of the mitochondrial genes cytochrome c oxidase subunit I (*COX1*), cytochrome b (*CYTB*) and 16S ribosomal RNA (*16S*), and the nuclear locus glycin transporter 1 (*GLYT1*); for the CAC analysis, we used the same markers, except *CYTB*. Primers were taken from Folmer *et al*. [27] and Costa & Amorim [28] for *COX1*; Palumbi *et al*. [29] for *CYTB* and *16S*; and, Li *et al*. [30] for *GLYT1*; the list of primers is given in S2 Table. Polymerase chain reaction (PCR) was performed in 30μl reaction mixtures containing 5x Green GoTaq Reaction Buffer (Promega), 3.2 mM MgCl_2_, 1 μM of each primer, 75 ng of total genomic DNA, 0.2 mM of each dNTP and 1U of Taq polymerase. The thermocycling profile included: 1 cycle of 4 minutes at 94°C; 35 cycles of 1 minute at 92°C, 1 minute at 47–63°C and 1 minute at 72°C; and 1 cycle of 4 minutes at 72°C. In all PCR reactions, negative controls without DNA were used to check contaminations. Amplified PCR products were purified using the Wizard SV Gel and PCR Clean-Up System (Promega). Sequencing reactions were made using the BigDye Terminator Cycle Sequencing Mix (Applied Biosystems). Cycle sequencing reactions were performed in 10 μl reaction volumes containing 1 μl BigDye 2.5, 1.55 μl 5x sequencing buffer (Applied Biosystems), 2 μl of the amplified products (10–40ng), and 2 μl primer. The thermocycling profile was: (1) 35 cycles of 10 seconds at 96°C, 5 seconds at 54°C and 4 minutes at 60°C. The sequencing reactions were purified and denatured and the samples were run on an ABI 3130 Genetic Analyzer. Sequences were edited using MEGA 7.0 [31].

### Phylogenetic analyses

Alignment for each gene dataset was conducted using Clustal W algorithm [32], implemented in MEGA 7.0 [31]; subsequently, DNA sequences were translated into amino acids residues to check premature stop codons or indels. The dataset of encoding protein genes was partitioned following each codon position. Jmodeltest 2.1.7 [33] was used to determine the best-fitting models of molecular evolution for each codon for all gene datasets based on Akaike information criteria (AIC); models of nucleotide substitution and partitions are provided in S3 Table.

Independent phylogenetic analyses for HAG and CAC were performed using GARLI v.2.0 [34] for Maximum Likelihood (ML) methods and Beast v.1.8. [35] for Bayesian inference (BI) and divergence date analyses of the concatenated dataset. ML searches for the best tree were performed in ten independent replications with at least 20,000 generations, since no topology improvement was observed by adding more generations; bootstrap values were calculated using 1,000 searches; all parameters between partitions except topology and branch lengths were unlinked. BI and divergence date analyses were performed using a lognormal uncorrelated relaxed clock model, with MCMC runs of 200 ×10^6^ generations, and a sampling frequency of 1000. The value of parameters of the analyses, convergence of the MCMC chains, sample size and the stationary phase of the chains were evaluated using Tracer v. 1.5 [36]. Consensus topology and posterior probabilities were obtained after applying a burn-in of the first 20% of the generated trees.

A Birth-Death speciation process for the tree prior [37] was used. In both analyses, a single calibration point was placed at the stem comprising the root of the tree, corresponding to the estimated age of the clade Cynolebiini (i.e., mean age of 20 Mya, minimum age of 16 Mya, standard deviation 1.2). This estimate was taken from a time-calibrated analysis involving different cyprinodontiform lineages [18], using as calibration points two fossil representatives of the European clade comprising Aphaniidae and Valenciidae, which includes the most rich and accurately determined fossil record among cyprinodontiforms. This analysis is temporally compatible to the analysis provided by [38] for acantomorph fishes based on 37 calibration points, showing similar age for cyprinodontiforms and main lineages.

**Fig 1:**
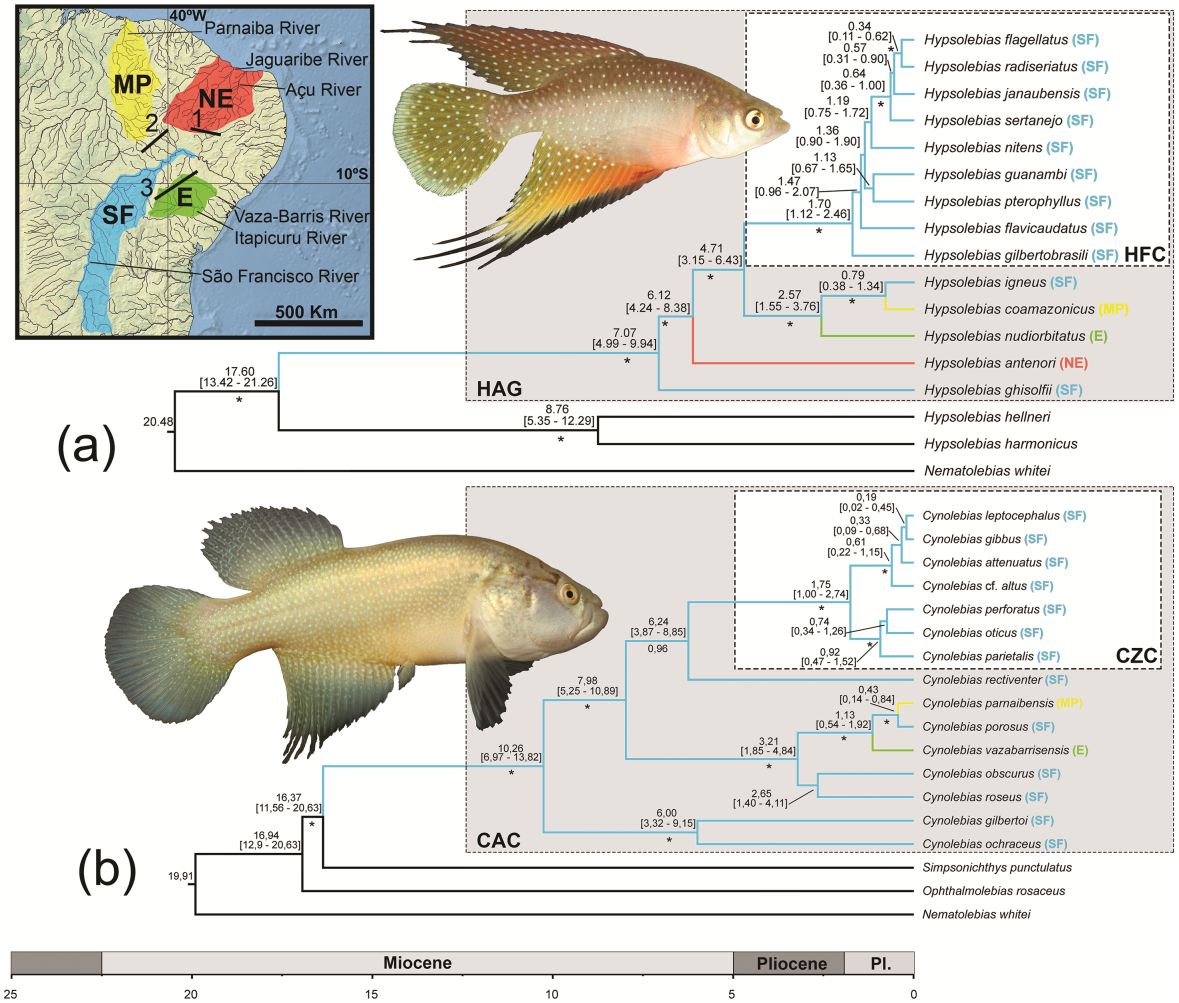
Time-scaled phylogeny obtained from the Bayesian analysis in BEAST. (a) Phylogenetic relationships among 14 species of the *Hypsolebias antenori* species group (HAG) and three out-group species; (b) Phylogenetic relationships among 15 species of the *Cynolebias* alpha-clade (CAC) and three out-group species; values above nodes are mean average ages of the nodes, followed below, between brackets, by the 95% highest posterior densities intervals for estimated ages; numbers below nodes are posterior probability values derived from the Bayesian analysis, where asterisks indicate maximum value; (CZC) indicates the *Cynolebias* zeta-clade and (HFC), the *Hypsolebias flavicaudatus* species complex. Areas delimitated in the map are ecoregions used in this study: Eastern (E); Maranhão-Piauí (MP); northeastern (NE); São Francisco (SF). Numbers in map indicate palaeogeographical events affecting diversification and present distribution of seasonal killifishes of the Caatinga: uplift of the Borborema massif separating NE ecoregion river basins from SF, Miocene (1); São Francisco River reconfiguration separating E ecoregion from SF Pliocene-Pleistocene (2); breaking of the São Francisco palaeocourse separating MP ecoregion from SF, Pleistocene (3). Photograph above is *Hypsolebias igneus* (Costa, 2000), below, *Cynolebias rectiventer* Costa, 2014.

Four areas of endemism were delimited following the Caatinga ecoregions delimitated as areas for fish endemism by Rosa *et al*. [39]: Eastern ecoregion: corresponds to the area occupied by the Vaza-barris, Itapicuru and Paraguaçu rivers; Maranhão-Piauí ecoregion: includes the areas of the Parnaíba River basin inserted in the Caatinga province; Northeastern ecoregion: comprises the Jaguaribe, Açu-Piranhas and other smaller basins of northeastern Brazil; São Francisco ecoregion: coincident with the São Francisco River basin in the Caatinga region (Fig 1).

## Results

Both BI and ML analyses generated identical trees (BI trees appear in Fig 1, ML trees in S1 Fig). Both HAG and CAC were recovered as monophyletic with high support and most included clades are highly supported. The phylogenetic analyses also recovered two diversified clades previously delimited by morphological characters, the *Hypsolebias flavicaudatus* complex [5] and the *Cynolebias* zeta-clade [4].

Divergence time estimates (Fig 1) indicate similar ages for HAG and CAC and included clades. Both clades originated during the Early Miocene, whereas species diversification took place at the Late Miocene. Synchronic divergences remarkably indicate similar vicariance events involving Caatinga ecoregions (Table 1), including a split between lineages endemic to the Maranhão-Parnaíba and the São Francisco ecoregions during the Pleistocene, and between lineages endemic to the East and the São Francisco ecoregions during the Late Pliocene–Early Pleistocene.

**Table 1:** Vicariance events involving Caatinga ecoregions supported in this study, their respective estimated ages and hypothesized associated palaeogeographical events.

Ecoregion split	Lineage split	Estimated age	Palaeogeographical event
SF/NE	*Hypsolebias antenori*	6.12 Mya; 95% HPD: 4.24–8.38 Mya	uplift of the Borborema massif separating NE river basins from SF
SF/E	*Hypsolebias nudiorbitatus*	2.57 Mya; 95% HPD: 1.55–3.76 Mya	São Francisco River reconfiguration separating E from SF
SF/E	*Cynolebias vazabarrisensis*	1.13 Mya; 95% HPD: 0.54–1.92 Mya	São Francisco River reconfiguration separating E from SF
SF/MP	*Hypsolebias coamazonicus*	0.79 Mya; 95% HPD: 0.38–1.34 Mya	breaking of the São Francisco palaeocourse separating MP from SF
SF/MP	*Cynolebias parnaibensis*	0.43 Mya; 95% HPD: 0.14–0.84 Mya	breaking of the São Francisco palaeocourse separating MP from SF

## Discussion

The analyses indicated that both HAG and CAC originated during the Miocene: HAG at 17.60 million years ago (Mya), ranging from 13.42 to 21.26 Mya following 95% highest posterior density interval (HPD); and CAC at 16.37 Mya (95% HPD: 11.56–20.63 Mya) (Fig 1). Few available information on palaeodrainages of northeastern Brazil indicates that the SFR had a different course during the Miocene [40, 41]. The main difference consisted in its lower section draining to north, being nearly coincident with part of the present Parnaíba River drainage. However, the distribution of most basal lineages of HAG and CAC in the SFR suggests that both groups originated in this palaeodrainage, then probably connected to other drainages of northeastern Brazil before the uplift of regional plateaus (see below).

In HAG, species diversification started between the Late Miocene and Early Pliocene (7.07 Mya; 95% HPD: 4.99–9.94 Mya). At this time earth was experiencing temperatures lower than in previous periods [42], which probably contributed to the expansion of semi-arid conditions in the present Caatinga region [13]. These conditions favoured dispersal and diversification of organisms adapted to SDTF, including endemic plants that reached high levels of diversification between the Late Miocene and Pliocene [1]. In CAC, a speciose clade including all species of *Cynolebias* endemic to the Caatinga except the morphologically divergent species pair, comprising *C*. *gilbertoi* Costa, 1998 and *C*. *ochraceus* Costa, 2014 [6], has its species diversification contemporary to HAG diversification (7.98 Mya; 95% HPD: 5.25–10.89 Mya). A temporally similar species diversification has also been reported for two groups of lizards endemic to the Caatinga [13, 15], supporting the hypothesis that intensification of drier periods beginning at the Miocene end [43] provided climatic conditions suitable for diversification of organisms adapted to life in the semi-arid Caatinga.

In a morphological analysis of *Cynolebias*, Costa [4] considered the sister-group relationships between *C*. *microphthalmus*, a species of CAC endemic to the northeastern ecoregion, and a generic clade endemic to the São Francisco and Eastern ecoregions as evidence of an old connection between the Jaguaribe and SFR basins. Costa *et al*. [20] based on the molecular analysis of a fragment of the mitochondrial gene cytb found *H*. *antenori*, a HAG species endemic to the northeastern ecoregion, sister to a clade endemic to all the other Caatinga ecoregions. This biogeographic pattern was interpreted as evidence of a vicariant event resulted from the uplift of the Borborema massif, which is the watershed between Jaguaribe-Piranhas basins and SFR basin, thus isolating the northeastern ecoregion from neighbouring areas. However, both Costa [4] and Costa *et al*. [20] analyses were not time-calibrated. The present study indicates that the divergence between *H*. *antenori* and that clade occurred in the Late Miocene (6.12 Mya; 95% HPD: 4.24–8.38 Mya), therefore congruent with the age of the final uplift of the Borborema massif in the Miocene [43]. Unfortunately, tissue suitable for DNA extraction of *C*. *microphthalmus*, the only species of CAC endemic to the northeastern ecoregion, was not available for this study (see Materials and methods above), not allowing us to estimate its phylogenetic position and age. However, a similar biogeographical pattern with coincident chronological divergence was reported for a saxicolous lizard group endemic to the Caatinga, in which a clade from the northeastern ecoregion diverged from a São Francisco clade during the Late Miocene [13].

The analyses indicated well-supported HAG and CAC clades containing species endemic to the eastern ecoregion (*H*. *nudiorbitatus* Costa, 2011 and *C*. *vazabarrisensis* Costa, 2001) sister to subclades containing one species endemic to the São Francisco ecoregion (*H*. *igneus* and *C*. *porosus* Steindachner, 1876) and one to the Maranhão-Piauí ecoregion (*H*. *coamazonicus* Costa, Amorim & Bragança, 2014 and *C*. *parnaibensis* Costa, Ramos, Alexandre & Ramos, 2010) (Fig 1). The clade including *H*. *nudiorbitatus*, *H*. *igneus*, and *H*. *coamazonicus* had already been recovered by Costa *et al*. [20], who mentioned the possibility of this biogeographic pattern to be a consequence of an old connection between SFR and the Parnaíba River, but no inference about timing of divergence was made. Our results support an age of 0.79 Mya (95% HPD: 0.38–1.34 Mya) for the split between *H*. *igneus* and *H*. *coamazonicus*, and 0.43 Mya (95% HPD: 0.14–0.84 Mya) for the split between *C*. *porosus* and *C*. *parnaibensis* (Fig 1). Considering the 95% highest posterior density intervals found for these divergences, both ranges are highly overlapped and indicated that these splits occurred during the Pleistocene. However, accurate data on the age of this river connection are not available. Former geological studies [40, 44] have supported a connection between SFR and the Parnaíba River basin until the Pliocene, but more recent studies [45] indicate that the present river configuration was reached only in the Pleistocene, during the Mindel glaciation, about 450.000 years ago. Our data thus indicated a nearly synchronous divergence isolating species of HAG and CAC endemic to the Parnaíba River basin (*i*.*e*., Maranhão-Piauí ecoregion), suggesting that some genetic flow persisted until the Pleistocene.

Two independent sister group relationships between species endemic to the eastern ecoregion (*H*. *nudiorbitatus* and *C*. *vazabarrisensis*) and clades encompassing species of the São Francisco and Maranhão-Piauí ecoregions highly suggest past connections between these three ecoregions. Werneck *et al*. [13] found evidence of an Early Pleistocene SFR palaeocourse south of its present lower section, through an area today occupied by river basins of the eastern ecoregion. Our analyses indicate that the divergence between *H*. *nudiorbitatus* and the clade comprising *H*. *igneus* and *H*. *coamazonicus* occurred between the Late Pliocene and Early Pleistocene (2.57 Mya; 95% HPD: 1.55–3.76 Mya), whereas the divergence between *C*. *vazabarrisensis* and the clade comprising *C*. *porosus* and *C*. *parnaibensis* occurred in the Pleistocene (1.13 Mya; 95% HPD: 0.54–1.92 Mya). Both divergences may be related to that palaeodrainage configuration, but geological data supporting this hypothesis are not available yet.

The analyses indicate a high synchronic species diversification in the *Hypsolebias flavicaudatus* complex sense Costa *et al*. [46] (hereafter HFC) and the *Cynolebias* zeta-clade sense Costa [4] (hereafter CZC) (Fig 1). Both clades are endemic to SFR basin and are broadly widespread in most parts of the basin, besides comprising numerous cryptic species [6, 46]. The analyses indicated that both clades highly diversified during the Pleistocene, starting at 1.70 Mya (95% HPD: 1.12–2.46 Mya) in HFC and 1.75 Mya (95% HPD: 1.00–2.74 Mya) in CZC (Fig 1). These data therefore support a major diversification during a period characterised by alternating dry and moist periods following glacial and interglacial periods of the Pleistocene. Based on pollen fossil records for an area of the Caatinga, Oliveira *et al*. [47] reported the occurrence of typical vegetation components of South American rain forests (*i*.*e*., Amazon and Atlantic Forest) during the Late Pleistocene, which were gradually substituted by Caatinga vegetational components just after Holocene climate optimum. Therefore, alternating periods of expansion and retraction of the Caatinga probably promoted diversification of these seasonal killifish clades during the Pleistocene.

## Conclusion

The methodological approach adopted in this study, consisting of independent time-calibrated phylogenetic molecular analyses of two clades of sympatric and geographically widespread seasonal fishes endemic to the Caatinga, allowed us to infer the evolutionary history of these clades and to correlate it to past geological and climatic factors. Our results consistently indicate that species diversification took place synchronically in both groups, as well as patterns of diversification are contemporary to other organisms adapted to life in the semi-arid Caatinga. These patterns are chronologically related to both Tertiary palaeogeographical reorganizations associated to continental drift and Quaternary climatic changes, corroborating the recent proposal that South American biodiversity has been continuously shaped between the Late Paleogene and Pleistocene.

## Supporting information

S1 Table List of taxa, GenBank accession numbers and coordinates of collecting sites. (DOC)

S2 Table Primers used in the analyses. (DOCX)

S3 Table Best-fitting models of molecular evolution. (DOCX)

S1 Fig Maximum Likelihood trees. (TIF)
