# Supplementary material for: Synchronic historical patterns of species diversification in seasonal aplocheiloid killifishes of the semi-arid Brazilian Caatinga
Source: PLoS One. 2018 Feb 16;13(2):e0193021. doi: 10.1371/journal.pone.0193021 (PMC5815601; doi:10.1371/journal.pone.0193021)
Supplement: S1 Table — (DOC) [file pone.0193021.s001.doc]

**S1 Table.** List of taxa, GenBank accession numbers and coordinates of collecting sites.

| Species | Cox1 | CytB | 16s | Glyt | Locality |
| --- | --- | --- | --- | --- | --- |
| *Cynolebias perforatus* Costa & Brasil, 1991 | | | |  |  |
|  | KY093024 | -------- | KY033502 | KY093052 | 15° 30' 00"S 44° 23' 18"W |
| *Cynolebias parietalis* Costa, 2014 | | | |  |  |
|  | KF311244 | -------- | MF663623 | MF663597 | 14° 55' 20"S 43° 29' 55"W |
| *Cynolebias attenuatus* Costa, 2001 | | | |  |  |
|  | KF311252 | -------- | MF663624 | MF663598 | 13° 15' 41"S 43° 31' 28"W |
| *Cynolebias ochraceus* Costa, 2014 | | | |  |  |
|  | KF311280 | -------- | -------- | MF663599 | 13° 15' 41"S 43° 31' 28"W |
| *Cynolebias gilbertoi* Costa, 1998 | | |  |  |  |
|  | KF311281 | -------- | KY033503 | KY093053 | 13° 34' 49"S 43° 22' 39"W |
| *Cynolebias* cf *altus* Costa, 2001 | | |  |  |  |
|  | KF311263 | -------- | MF663625 | MF663600 | 11° 28' 03"S 43° 17' 10"W |
| *Cynolebias porosus* Steindachner, 1876 | | | |  |  |
|  | KF311289 | -------- | KY033506 | KY093056 | 08° 52' 36"S 39° 55' 24"W |
| *Cynolebias roseus* Costa, 2014 | | |  |  |  |
|  | KF311291 | -------- | MF663626 | MF663601 | 10° 58' 28"S 43° 03' 00"W |
| *Cynolebias leptocephalus* Costa & Brasil, 1993 | | | | |  |
|  | KF311276 | -------- | MF663627 | MF663602 | 14° 12' 54"S 42° 50' 22"W |
| *Cynolebias oticus* Costa, 2014 | | |  |  |  |
|  | KF311297 | -------- | MF663628 | MF663603 | 14° 33' 29"S 42° 42' 07"W |
| *Cynolebias obscurus* Costa, 2014 | | | |  |  |
|  | KF311299 | -------- | MF663629 | MF663604 | 13° 03' 10"S 42° 36' 57"W |
| *Cynolebias parnaibensis* Costa, Ramos, Alexandre & Ramos, 2010 | | | | | |
|  | KF311304 | -------- | MF663630 | MF663605 | 08° 00' 54"S 41° 25' 26"W |
| *Cynolebias vazabarrisensis* Costa, 2001 | | | |  |  |
|  | KF311305 | -------- | KY033504 | KY093054 | 10° 19' 34"S 39° 03' 44"W |
| *Cynolebias rectiventer*  Costa, 2014 | | | |  |  |
|  | KF311285 | -------- | KY033505 | KY093055 | 10° 43' 19"S 42° 20' 31"W |
| *Cynolebias gibbus* Costa, 2001 | | |  |  |  |
|  | KF311272 | -------- | MF663631 | MF663606 | 13° 00' 46"S 43° 28' 50"W |
| *Hypsolebias harmonicus* (Costa, 2010) | | | |  |  |
|  | JQ612736 | JQ612736 | KY033490 | KY093043 | 13° 15' 42"S 43° 31' 28"W |
| *Hypsolebias hellneri* (Berkenkamp, 1993) | | | |  |  |
|  | KY033499 | JQ612735 | KY033499 | KJ844634 | 15° 04' 50"S 44° 04' 40"W |
| *Hypsolebias ghisolfii* (Costa, Cyrino & Nielsen, 1996) | | | | |  |
|  | HQ833478 | JQ612737 | KY033489 | KY093042 | 14° 40' 09"S 42° 41' 00"W |
| *Hypsolebias igneus* (Costa, 2000) | | | |  |  |
|  | HQ833482 | JQ612741 | KY033491 | KY093044 | 11° 27' 19"S 43° 16' 43"W |
| *Hypsolebias flagellatus* (Costa, 2003) | | | |  |  |
|  | HQ833481 | JQ612744 | MF663632 | MF663607 | 13° 15' 41"S 43° 31' 28"W |
| *Hypsolebias sertanejo* Costa, 2012 | | | |  |  |
|  | JQ612753 | JQ612757 | KY033492 | MF663608 | 15° 30' 00"S 44° 23' 18"W |
| *Hypsolebias radiseriatus* Costa, 2012 | | | |  |  |
|  | MF663617 | JQ612751 | MF663633 | MF663609 | 14° 55' 20"S 43° 29' 55"W |
| *Hypsolebias pterophyllus* Costa, 2012 | | | |  |  |
|  | MF663618 | JQ612747 | MF663634 | MF663610 | 12° 28' 52"S 43° 11' 28"W |
| *Hypsolebias guanambi* Costa & Amorim, 2011 | | | | |  |
|  | HQ833486 | JQ612768 | -------- | MF663611 | 14° 13' 41"S 42° 55' 11"W |
| *Hypsolebias janaubensis* (Costa, 2006) | | | |  |  |
|  | HQ833487 | JQ612772 | MF663635 | MF663612 | 15° 47' 57"S 43° 19' 17"W |
| *Hyposlebias gilbertobrasili* Costa, 2012 | | | |  |  |
|  | MF663619 | JQ612771 | MF663636 | MF663613 | 13° 34' 49"S 43° 22' 39"W |
| *Hypsolebias flavicaudatus* (Costa & Brasil, 1990) | | | | |  |
|  | HQ833492 | JQ612777 | KY033494 | KY093046 | 08° 52' 36"S 39° 55' 25"W |
| *Hypsolebias nitens* Costa, 2012 | | |  |  |  |
|  | MF663620 | JQ612778 | MF663637 | MF663614 | 10° 43' 21"S 42° 20' 31"W |
| *Hypsolebias nudiorbitatus* Costa, 2011 | | | |  |  |
|  | MF663621 | KF311241 | KY033495 | KY093047 | 10° 43' 32"S 40° 06' 14"W |
| *Hypsolebias coamazonicus* Costa, Amorim & Bragança, 2014 | | | | | |
|  | MF663622 | KF311235 | MF663638 | MF663615 | 03° 05' 87"S 41° 53' 56"W |
| *Hypsolebias antenori* (Tulipano, 1973) | | | |  |  |
|  | KU594343 | KF311231 | KY033493 | KY093045 | 05° 13' 41"S 38° 08' 06"W |
| *Hypsolebias longignatus* (Costa, 2008) | | | |  |  |
|  | KY093022 | MF663616 | KY033500 | -------- | 03° 53' 48"S 38° 24' 17"W |
| *Nematolebias whitei* (Myers, 1942) | | | |  |  |
|  | KT590060 | KF311321 | KT590049 | KT590078 | 22° 43' 59"S 42° 02' 29"W |
| *Ophthalmolebias rosaceus* (Costa, Nielsen & de Luca, 2001) | | | | | |
|  | KY093035 | -------- | KY033517 | KY093065 | 15° 31' 23"S 39° 36' 02"W |
| *Simpsonichthys punctulatus* Costa & Brasil, 2007 | | | | |  |
|  | KY093037 | -------- | KY033519 | KY093066 | 15° 33' 01"S 47° 10' 33"W |
