# Supplementary material for: Synchronic historical patterns of species diversification in seasonal aplocheiloid killifishes of the semi-arid Brazilian Caatinga
Source: PLoS One. 2018 Feb 16;13(2):e0193021. doi: 10.1371/journal.pone.0193021 (PMC5815601; doi:10.1371/journal.pone.0193021)
Supplement: S2 Table — (DOCX) [file pone.0193021.s002.docx]

**S2 Table.** Primers used in the analyses.

| Gen | Primer | Sequence | Reference |
| --- | --- | --- | --- |
| COX1 | LCO1490 | GGT CAA CAA ATC ATA AAG ATA TTG G | Folmer *et al.*, 1994 |
|  | Cox1R | GGY TCT TCR AAR GTG TGA TAS G | Costa & Amorim, 2011 |
| CYTB | CB3-H | TGA CCT GAA RAA CCA YCG TTG | Palumbi *et al*., 2002 |
|  | Gludg-L | GGC AAA GAG AAA RTA TCA TTC | Palumbi *et al*., 2002 |
| 16s | 16sar-L | CGC CTG TTT AYC AAA AAC AT | Palumbi *et al*., 2002 |
|  | 16sbr-H | CCG GTC TGA ACT CAG ATC ACG T | Palumbi *et al.*, 2002 |
|  | R16sn | GGA TGT CCT GAT CCA ACA TCG AGG TCG TA | Costa *et al*., 2016 |
| GLYT | Glyt_F577 | ACATGGTACCAGTATGGCTTTGT | Li *et al*., 2007 |
|  | Glyt_R1562 | CCCAAGAGGTTCTTGTTRAAGAT | Li *et al*., 2007 |
