# Supplementary material for: Synchronic historical patterns of species diversification in seasonal aplocheiloid killifishes of the semi-arid Brazilian Caatinga
Source: PLoS One. 2018 Feb 16;13(2):e0193021. doi: 10.1371/journal.pone.0193021 (PMC5815601; doi:10.1371/journal.pone.0193021)
Supplement: S3 Table — (DOCX) [file pone.0193021.s003.docx]

**S3 Table.** Best-fitting models of molecular evolution.

|  |  |  |  |  |
| --- | --- | --- | --- | --- |
| Taxa | Gene | Codon position | Base pairs | Evolutive model |
| *Hypsolebias* | 16S |  | 577 | GTR+G |
|  | COX1 | COX1_1st | 450 | GTR+G |
|  |  | COX1_2nd | 451 | F81 |
|  |  | COX1_3rd | 451 | GTR+G |
|  | CYTB | CYTB_1st | 154 | K80+I |
|  |  | CYTB_2nd | 154 | GTR+G |
|  |  | CYTB_3rd | 153 | GTR+G |
|  | GLYT | GLYT_1st | 256 | F81 |
|  |  | GLYT_2nd | 256 | HKY |
|  |  | GLYT_3rd | 256 | K80 |
| *Cynolebias* | 16S |  | 574 | GTR+G |
|  | COX1 | COX1_1st | 225 | SYM+G |
|  |  | COX1_2nd | 225 | HKY+I |
|  |  | COX1_3rd | 225 | GTR+G |
|  | GLYT | GLYT_1st | 328 | GTR |
|  |  | GLYT_2nd | 328 | HKY+I |
|  |  | GLYT_3rd | 327 | GTR |
