# Supplementary figures and images for: Synchronic historical patterns of species diversification in seasonal aplocheiloid killifishes of the semi-arid Brazilian Caatinga
Source: PLoS One. 2018 Feb 16;13(2):e0193021. doi: 10.1371/journal.pone.0193021 (PMC5815601; doi:10.1371/journal.pone.0193021)

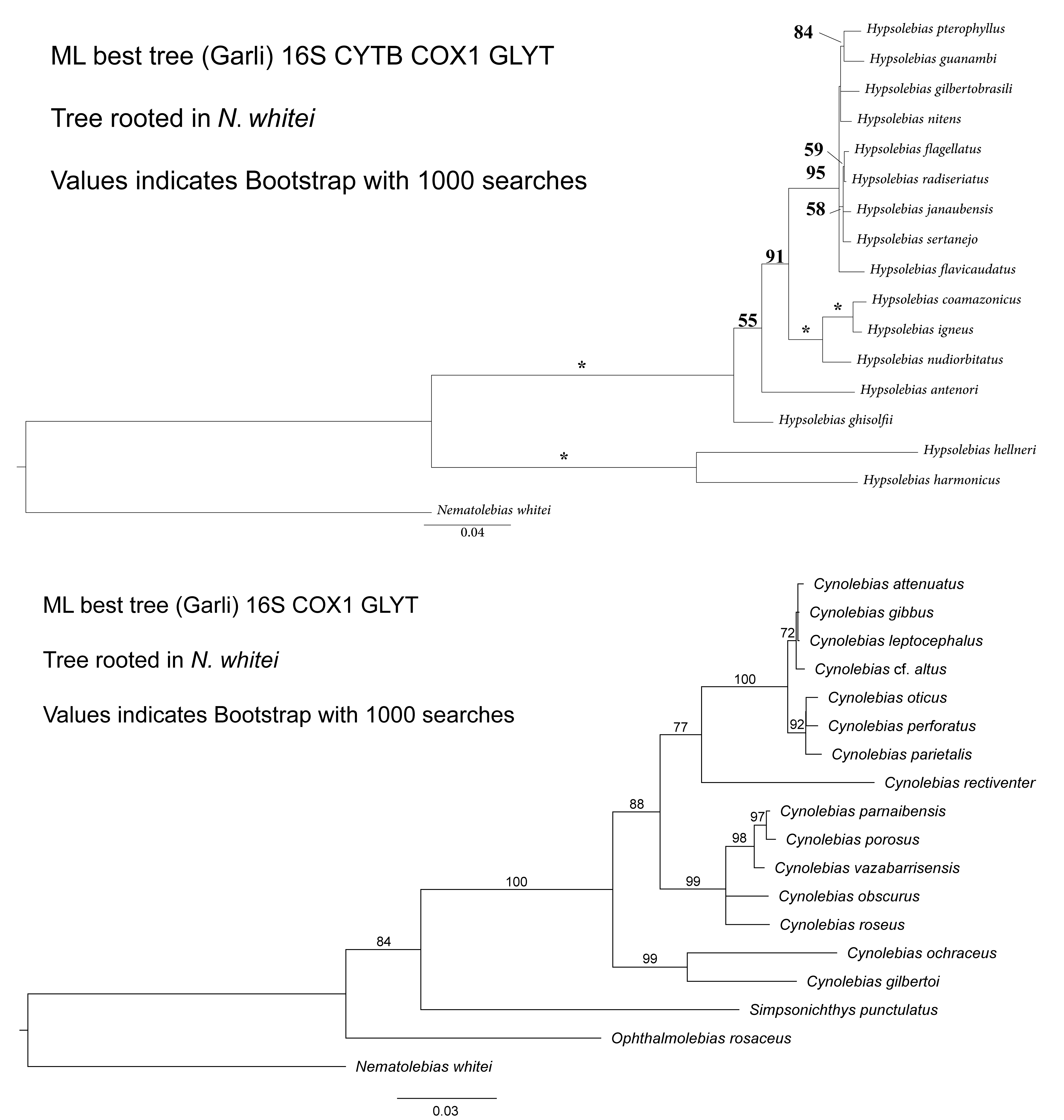

Supplement: S1 Fig — (TIF) [file pone.0193021.s004.tif]
